# Ambassadors of Clinical Psychology and Psychological Treatment

**DOI:** 10.32872/cpe.8545

**Published:** 2022-03-31

**Authors:** Claudi Bockting, Winfried Rief

**Affiliations:** 1 Department of Psychiatry, Amsterdam University Medical Centers, location AMC, University of Amsterdam, Amsterdam, The Netherlands; 2 Division of Clinical Psychology and Psychotherapy, Department of Psychology, Philipps-University of Marburg, Marburg, Germany

The European Association of Clinical Psychology and Psychological Treatment (EACLIPT) board has decided to nominate Ambassadors of Clinical Psychology and Psychological Treatment. Ambassadors are selected according to their achievements for our field, but also according to the perspectives for further fostering the visibility and impact of clinical psychology. The typical profile of our ambassadors is the high quality of translational research. Ambassadors commit to show their support for the association and its mission. 

We are proud that two extremely well-known colleagues confirmed to become ambassadors for EACLIPT: Paul Emmelkamp and Peter Fonagy, and they will be introduced in this issue. We are extending this list and will announce it in future issues of CPE, and we promise to consider gender and diversity issues. Congratulations to Paul Emmelkamp and to Peter Fonagy, and to all of us because we have tremendous personalities in our group.



**Claudi Bockting**
*(President of EACLIPT)*
**Winfried Rief**
*(Editor of CPE)*



